# Engineered mRNA Nanoparticle Platforms for Respiratory Mucosal Delivery

**DOI:** 10.3390/vaccines14070596

**Published:** 2026-07-04

**Authors:** Rui Jin, Bao-Zhong Wang, Wandi Zhu

**Affiliations:** 1 Emory Institute for Drug Development, Emory University, Atlanta, GA 30322, USA; jin.rui@emory.edu; 2 Center for Inflammation, Immunity & Infection, Institute for Biomedical Sciences, Georgia State University, Atlanta, GA 30303, USA; bwang23@gsu.edu

**Keywords:** mRNA, lipid nanoparticle, polymer-based nanoparticle, extracellular vesicle, hybrid nanoparticle, mucosal immune response, PEG, adjuvant

## Abstract

Respiratory mucosal vaccination can induce robust humoral and cellular immune responses, as well as effective mucosal immunity at the primary site of pathogen entry, and has been shown to provide superior protection against respiratory viral infections compared with traditional approaches. Among current vaccine technologies, mRNA vaccines offer unique advantages, including rapid development, flexible antigen design, and potent immunogenicity. However, efficient mucosal delivery of mRNA remains challenging due to biological barriers within the respiratory tract, including mucus clearance, limited cellular uptake, and instability during aerosolization. Furthermore, mRNA formulations intended for respiratory mucosal delivery require more stringent safety and tolerability profiles. Recent advances in nanoparticle engineering have accelerated the development of mRNA delivery systems optimized for respiratory mucosal immunization. This review aims to evaluate how nanoparticle engineering strategies can overcome respiratory mucosal barriers and improve the safety, stability, delivery efficiency, extrahepatic expression, and immunogenicity of mRNA vaccines and therapeutics. We summarize recent progress in engineered mRNA nanoparticle platforms for respiratory mucosal immunity, encompassing modified lipid nanoparticles (LNPs), polymer-based mRNA nanoparticles, and hybrid nanoparticle systems, including lipid-inorganic, polymeric hybrid, and lipid-extracellular vesicle (EV) nanoparticles. We further discuss optimization strategies for mucosal mRNA delivery, including the incorporation of appropriate adjuvants, the development of polyethylene glycol (PEG) alternatives, and advanced delivery approaches. Finally, we highlight current challenges and future directions for the rational design of next-generation mRNA nanoparticle platforms that can induce durable and broadly protective mucosal immunity against respiratory viral infections.

## 1. Introduction

Respiratory viral infections, including SARS-CoV-2, influenza, and respiratory syncytial virus (RSV), remain major global health threats that cause substantial morbidity and mortality worldwide. During July 2024–June 2025, COVID-19 was associated with an estimated 290,000–450,000 hospitalizations and 34,000–53,000 deaths in the United States, while RSV was associated with 190,000–350,000 hospitalizations and 10,000–23,000 deaths [1]. The 2024/2025 influenza season was marked by approximately 51 million influenza-related illnesses with 710,000 hospitalizations and 45,000 deaths, representing the first high severity season since 2017–2018 [2].

The continued global burden of respiratory viral infections underscores the urgent need for next-generation vaccines capable of responding rapidly to evolving and newly emerging pathogens. The rapid development and clinical success of mRNA lipid nanoparticle (LNP) vaccines during the COVID-19 pandemic demonstrated the transformative potential of this platform for combating emerging infectious diseases [3,4,5]. Compared with conventional vaccine platforms, mRNA vaccines offer several advantages, including rapid antigen design, scalable manufacturing, an acceptable safety profile, and the ability to induce both humoral and cellular immune responses [6]. In addition, advances in nucleoside modification, purification, and LNP technologies have markedly improved mRNA stability, translational efficiency, and in vivo delivery [7,8].

Despite these advances, the route of administration remains one of key factors influencing the induction of protective immune responses against respiratory pathogens. Currently approved intramuscular mRNA vaccines are highly effective at preventing severe disease but induce only limited mucosal immunity within the respiratory tract, which is the primary site of entry and replication for respiratory viruses. Effective mucosal immunity is essential for restricting viral infection, reducing transmission, and providing rapid frontline protection [9,10]. The respiratory mucosal immune system comprises epithelial barriers, mucus layers, secretory immunoglobulin A (sIgA), innate immune populations, and tissue-resident memory T and B cells (T_RM_ and B_RM_), which collectively coordinate local immune defense. In particular, mucosal sIgA and tissue-resident cells may rapidly respond to respiratory viruses at the site of infection [11,12]. Growing evidence suggests that parenteral administration of an mRNA vaccine alone cannot efficiently induce these localized immune responses, highlighting the need to develop mucosal vaccination strategies capable of eliciting robust mucosal immunity in the respiratory tract [13,14].

Recent advances in nanotechnology have facilitated the development of mRNA nanoparticle systems capable of delivering RNA via the respiratory tract, promoting local antigen expression in the lung [15,16]. Despite the advances, the development of effective nasal and pulmonary mRNA vaccines remains challenging because the respiratory tract possesses multiple biological barriers that limit nanoparticle transport and intracellular delivery. Among these, the mucus barrier represents a major obstacle to efficient mucosal mRNA delivery. Airway mucus is a highly viscoelastic gel, composed of mucins, lipids, salts, and antimicrobial proteins, that traps and clears inhaled particles and pathogens [17]. Increasing efforts have been made to develop RNA nanoparticle systems to overcome barriers and promote antigen expression in the respiratory tract [18,19], highlighting their potential to enhance the efficacy of mRNA vaccines for mucosal immunization.

This narrative review summarizes recent advances in engineered mRNA nanoparticle platforms for respiratory mucosal delivery and vaccination. We focus specifically on nanoparticle systems shown to overcome key respiratory barriers, including modified lipid nanoparticles (LNPs), polymer-based nanoparticles, and hybrid nanoparticle platforms incorporating lipid, polymeric, inorganic, or extracellular vesicle components. The literature reviewed was drawn primarily from the recent five years, a period marked by the rapid expansion of mRNA vaccine technologies following the COVID-19 pandemic and the emergence of next-generation respiratory RNA delivery systems. Given the diversity of nanoparticle compositions, delivery routes, experimental models, and study objectives, a narrative than systemic review format was considered to provide a comprehensive overview of current engineering strategies, recent advances, and future directions in the field. Because relatively limited studies have directly evaluated respiratory mucosal mRNA vaccination, this review also includes nanoparticle platforms that demonstrate efficient delivery, tropism, or protein expression in respiratory tissues, as these characteristics provide important insight into nanoparticle design and may facilitate the future development of respiratory mucosal vaccines and therapeutics.

## 2. Modified mRNA Lipid Nanoparticles (LNPs)

LNPs remain the most clinically advanced platform for mRNA delivery and have been extensively investigated and optimized for vaccine formulations. The clinical success of the Pfizer-BioNTech and Moderna COVID-19 vaccines established ionizable LNPs as the leading platform for mRNA delivery. These formulations typically contain an ionizable lipid, helper lipid, cholesterol, and PEG-lipid, which collectively facilitate mRNA encapsulation, cellular uptake, and endosomal escape following intramuscular administration. However, compared with parenteral administration, the development of pulmonary and intranasal mRNA-LNPs inducing tolerable and protective mucosal immunity in respiratory system is more challenging but of great importance in fighting against emerging respiratory virus epidemics and pandemics.

Besides the structure and purity of cargo mRNA, the lipid composition can be modified to improve mucus penetration, endosomal escape, and tolerability. Clinically approved ionizable lipids, including ALC-0315, SM-102, and DLin-MC3-DMA, have been incorporated into mRNA-LNPs, either alone or in combination with a permanent cationic lipid such as DOTAP and DOTMA to modulate the lipid composition. While these formulations effectively transfected both epithelial and immune cells in the lungs after intranasal delivery, the respiratory mucosa demonstrated limited immunogenicity. Nevertheless, mRNA-LNP vaccination can effectively recall immune responses in draining lymphoid tissues following mucosal administration [20]. Moreover, incorporation of additional cationic lipid components has been shown to alter tissue tropism and redirect mRNA delivery toward extrahepatic tissues. Several studies reported that adding a supplemental cationic lipid component to traditional lipid formulations significantly increased lung-selective delivery [20,21,22]. In another method, the replacement of standard helper lipids with neutral lipids, anionic lipids and cationic lipids, such as DOPE, sphingomyelin, ceramide, phosphatidylserine (PS), and DOTAP, has shown successful non-hepatocellular mRNA delivery while maintaining a four-component lipid formulation [21]. In another study, hybrid ionizable lipid-like nanoparticles (iLLNs) were developed by combining ionizable and cationic lipids at optimized ratios. The study demonstrated that modulation of the ratio of ionizable to cationic lipids can tune the pKa of iLLNs to match the nasal environment (pH 5.5–6.5), enabling the formation of near-neutral PEGylated “muco-inert” surfaces that minimize adhesive interactions with mucus while enhancing mucus penetration. Notably, their liquid-core structure could further enhance particle deformability and facilitate efficient transport across the mucus layer. In a mice study, optimized iLLN formulations achieved approximately 60-fold higher reporter gene expression in the nasal cavity after intranasal administration [23]. The triolein and *β*-sitosterol contained in iLLNs lipids formulation might contribute to the improved tolerability [24,25].

The presence of negatively charged and hydrophobic domains within airway mucus drives adhesive interaction between mucin and uncoated nanoparticles. To mitigate this, hydrophilic non-ionic polymers such as poly(ethylene glycol) (PEG), poly(2-alkyl-2-oxazolines) (POx), and poly(vinyl alcohol) (PVA) have been widely applied as protective surface coatings. For example, densely PEGylated polymeric nanoparticles exhibited rapid penetration into the mucus and enhanced pulmonary drug delivery [26,27,28,29]. However, it was also observed that increasing PEG-lipid density beyond approximately 5% improved mucus permeability yet simultaneously compromised transfection efficiency within the respiratory tract [26]. Cholesterol structure was found to critically influence biodistribution and cellular uptake, likely through its effects on endocytic pathways. Notably, cationic cholesterol-containing LNPs exhibited distinct delivery characteristics relative to those formulations with conventional cationic helper lipids. The combination of cationic cholesterol with helper lipids enabled efficient mRNA delivery across multiple lung cell populations, including epithelial and stem-like cells, as well as cardiac tissues following intravenous administration [30]. Collectively, these findings indicate that rational lipid engineering holds considerable promise for achieving targeted extrahepatic delivery. In addition, substituting cholesterol with β-sitosterol has been proposed as a strategy to evade recognition by endosomal membrane cholesterol transporters, thereby enhancing LNP endosomal escape efficiency [23,29].

Overall, current modified LNP strategies demonstrate distinct design priorities. Ionizable LNPs provide robust transfection efficiency and favorable clinical translatability but often exhibit limited mucosal performance in the respiratory tract. In contrast, incorporation of cationic lipids or cholesterol derivatives can enhance lung targeting and cellular uptake, although these modifications may increase formulation-dependent inflammatory responses. Similarly, surface PEGylation improves mucus penetration but may reduce cellular uptake at higher densities. These findings highlight that no single lipid modification simultaneously optimizes delivery efficiency, mucus penetration, and tolerability, underscoring the need for balanced and application-specific LNP design.

Ionizable LNPs remain the most clinically advanced platform for mRNA delivery due to their high encapsulation efficiency and established clinical track record. However, achieving effective pulmonary delivery remains challenging because formulations optimized for intracellular delivery often exhibit increased local inflammatory responses and tolerability concerns, particularly following intranasal administration and when cationic lipids are incorporated. Notably, a recent study by Moderna demonstrated that intranasal delivery of a SARS-CoV-2 mRNA-LNP vaccine conferred protective efficacy in a hamster model, suggesting that continued formulation refinement may address existing safety and tolerability challenges [31].

## 3. Polymer-Based mRNA Nanoparticles

Polymer-based nanoparticles represent an alternative platform for mucosal mRNA delivery and may offer greater flexibility for respiratory administration. Compared with conventional cationic LNPs, polymeric systems are increasingly explored for their potential to reduce cytotoxicity and inflammatory responses associated with intranasal vaccination. Cationic polymers can condense mRNA into polyplexes, protecting RNA from nuclease-related degradation and facilitating cellular internalization via endocytosis. A diverse range of modified cationic polymers has been synthesized, screened and investigated for mRNA nanoparticle applications.

Polyethyleneimine (PEI) is a well-characterized synthetic cationic polymer for nucleic acid delivery [32]. Its high charge density, water solubility, and low intrinsic immunogenicity render PEI an attractive scaffold for RNA nanoparticle engineering. Notably, a self-assembled PEI-complexed self-amplifying RNA (saRNA) recently demonstrated superior biological activity relative to earlier saRNA/PEI formulations in both in vitro and in vivo settings. When encoding influenza hemagglutinin (HA), PEI-complexed saRNA successfully elicited antigen-specific immune responses against influenza virus in murine models [32]. Despite these encouraging results, no PEI-based product has yet reached commercialization, largely due to the inherent difficulty of balancing therapeutic efficacy with acceptable tolerability.

Poly(β-amino esters) (PBAEs), a class of degradable polymers, have demonstrated capacity for systemic mRNA delivery to the lungs in mice when co-formulated with PEG-lipid conjugates [33]. Inhaled hyperbranched PBAE (hPBAE) mRNA polyplexes produced consistent pulmonary protein expression with minimal observed toxicity [34]. In a notable application, nebulized mRNA-encoding CRISPR-associated protein 13a (Cas13a) co-delivered with a guide RNA via a PBAE-based carrier, significantly reduced SARS-CoV-2 replication in both mice and hamsters [35]. Degradable polymer–lipid nanoparticles can be further optimized through tailored polymer synthesis and formulation design to facilitate selective lung delivery in vivo [36,37]. A next-generation candidate, P76, a poly(β-amino-thio-ester) (PBATE), demonstrated potent mRNA delivery across multiple animal models, including mice, hamsters, ferrets, cows and rhesus macaques. P76 also exhibited a favorable safety and tolerability profile, achieved superior expression levels relative to prior nebulized PBAE candidates, and enabled fourfold dose sparing compared with earlier PBAE in a Cas13a efficacy study against SARS-CoV-2 in hamsters [38]. A high-throughput image-based screening assay further revealed that PBAE nanoparticles with varying hydrophobic monomer compositions supported safe and efficacious mRNA expression across multiple tissues following intravenous injection in mice, with particularly high pulmonary expression observed across most formulations [39]. Additionally, co-delivery of Toll-like receptor agonist adjuvants alongside antigen-encoding mRNA via engineered bioreducible lipophilic PBAEs induced robust antigen-specific CD8^+^ T-cell responses [40]. Collectively, these findings highlight the promising application of PBAE-based nanoparticles for RNA respiratory delivery and mucosal vaccination, with PBAE-mRNA nanoparticles capable of eliciting both robust humoral and cellular responses in the respiratory tract.

Biodegradable poly(amine-co-ester) (PACE) nanoparticles have similarly been developed as platforms for mucosal mRNA vaccination. Intranasal administration of SARS-CoV-2 mRNA encapsulated in PACE nanoparticles induced protective mucosal immunity in rodents. Optimization of amine-containing end groups enhanced endosomal escape, and modifications to PEG content further improved mRNA delivery efficiency. Importantly, PACE-mRNA formulations demonstrated a favorable safety profile, with no elevation of liver enzymes or evidence of lung tissue damage in mice. Intratracheally administered PACE-mRNA complexes were efficiently internalized by both epithelial cells and antigen-presenting cells within the lungs, and the vaccination conferred protective immunity in hamsters [41]. In a follow-up investigation, the same group developed a red blood cell (RBC) hitchhiking strategy, whereby adsorption of PACE nanoparticles onto RBCs prior to administration was shown to modulate the nanoparticle biodistribution. The optimized approach enhanced delivery of PACE60 nanoparticle to murine visceral organs, with preferential accumulation in the lung [42]. Separately, hyperbranched PACE-based nanoparticles (HBPA-E) exhibited significantly greater in vivo pulmonary and intranasal delivery efficiency than their linear PACE counterparts. Notably, HBPA-E-formulated mRNA was amenable to lyophilization, making it a promising candidate for the practical clinical translations of mRNA therapeutics [43]. Therefore, these findings underscore the promise of the PACE platform for respiratory mucosal vaccination, though validation in large-animal models and clinical studies remains necessary to confirm its translational utility.

Although significant progress has been achieved with polymer-based nanoparticles for nasal mRNA delivery, generating robust and durable protective immune responses remains a critical challenge [38,41]. Compared with LNPs, polymer-based nanoparticles offer greater structural flexibility and opportunities for stimulus-responsive design. Nevertheless, highly cationic polymers that enhance cellular uptake may also induce cytotoxicity and inflammatory responses, limiting their clinical translation. Continued development of biodegradable and charge-modulated polymers may help improve their safety profile.

Overall, polymer-based mRNA delivery systems exhibit distinct performance profiles depending on polymer chemistry and structural design. PEI provides strong nucleic acid condensation and high transfection efficiency; however, its application is limited by cytotoxicity concerns. PBAE-based systems offer improved biodegradability and tunable in vivo performance, with emerging formulations achieving efficient pulmonary delivery and favorable safety profiles in preclinical models. In contrast to the extensive optimization and evaluation of PBAE-based platforms for in vivo mRNA delivery, PACE nanoparticles represent a newer class of degradable polymer delivery systems that enable efficient mRNA delivery to the lung and have also been explored as an intranasal SARS-CoV-2 vaccine capable of inducing protective immunity against viral infection. Further research holds promise for optimizing PACE-like polymers for mRNA mucosal vaccine applications. However, direct comparison across polymer systems is limited by differences in experimental models, administration routes, and assay endpoints, making cross-study evaluation challenging.

## 4. Hybrid RNA Nanoparticle Platforms

Beyond polymeric and lipid-based carriers, hybrid nanoparticle systems have emerged as a compelling strategy for RNA delivery, integrating the complementary strengths of distinct materials to overcome the inherent limitations of individual platforms.

### 4.1. Replicon RNA/Lipid-Inorganic Hybrid Nanoparticles

Lipid-inorganic nanoparticles (LIONs) are highly stable cationic oil-in-water emulsions in which superparamagnetic iron oxide (Fe_3_O_4_) nanoparticles (SPIO) are embedded in a hydrophobic oil phase composed of squalene, Span60 and DOTAP. Replicon RNA (repRNA) can be assembled on the surface of LION through electrostatic interaction with the cationic DOTAP. Intramuscular vaccination with a SARS-CoV-2 spike-encoding repRNA/LION nanoparticle vaccine elicited robust humoral and T-cell responses in both mice and nonhuman primates [44]. Furthermore, LION-encapsulated repRNA encoding multi-component antigens induced strong antibody responses with reduced systemic inflammation relative to conventional LNPs in pigtail macaques [45], positioning repRNA/LION formulations as a potential alternative to mitigate severe inflammatory responses, enhance tolerability, and broaden the applicability of repRNA delivery. LION-based formulations have advanced into Phase I clinical trials (NCT04844268 and NCT05132907) to evaluate the tolerability and immunogenicity of HDT-301 vaccine which is a LIONs formulated with repRNA encoding SARS-CoV-2 S antigen, in both vaccine-naïve participants and individuals who had previously received a SARS-CoV-2 vaccine. Results from these trials are anticipated to inform the broader application of RNA nanoparticle platforms in human vaccination. A recent study further demonstrated that lyophilization of LION/repRNA with 10% sucrose as a cytoprotectant adequately preserved in vitro antigen expression and elicited antigen-specific antibody responses in mice comparable to those induced by liquid LION/repRNA following intramuscular immunization. Notably, lyophilized LION/repRNA vaccines retained stability for at least 1 year at 2–8 °C [46], making them attractive candidates for future evaluation in the context of pulmonary delivery.

### 4.2. Lipid–Polymer Hybrid Nanoparticles

Lipid–polymer hybrid nanoparticles represent a promising approach to mRNA delivery, integrating biodegradable polymeric cores with lipid components to simultaneously leverage stability, controlled release and transfection efficiency. Poly(lactic-co-glycolic acid) (PLGA) is an FDA-approved biodegradable polymer widely used in sustained-release formulations [47] and has been extensively investigated as a nucleic acid delivery vehicle owing to its capacity to enhance endosomal escape and facilitate mRNA delivery to antigen-presenting cells (APCs) [48,49]. The inherent hydrophobicity of standard PLGA requires supplementation with polymer or lipids, such as poly(vinyl alcohol) (PVA) or PEG-lipid, to maintain surface-active properties through their interaction with hydrophobic PLGA core [50]. Co-formulation of PLGA with cationic polymers, including PEI and poly-L-lysine (PLL), has been shown to further improve RNA delivery efficiency [51,52]. A hybrid system incorporating PLGA and PBAE was developed to enhance pulmonary mRNA delivery. The PLGA/PBAE hybrid nanoparticles demonstrated the ability to penetrate the mucus barrier and transfect mRNA in ex vivo human lung tissues. Following vibrating-mesh nebulization, the formulation retained superior activity relative to SM102 LNPs, supporting PLGA/PBAE as a viable platform for inhaled mRNA vaccine delivery [49].

PLGA has also been co-formulated with conventional lipid components for mRNA fabrication and delivery. PLGA4-incorporated TT3 lipid–polymer hybrid nanoparticles demonstrated significantly enhanced mRNA delivery efficiency compared with LNPs composed of TT3 lipid, DOPE, cholesterol, and DMG-PEG2000 [53]. Similarly, SARS-CoV-2 spike mRNA-loaded lipid–polymer nanoparticles based on PLGA, the ionizable cationic lipid C12-200, DOPE and DMPE-PEG2000, induced robust spike-specific antibody and CD8^+^ T-cell responses in mice, and conferred protection against SARS-CoV-2 infection in Syrian golden hamsters [54].

Beyond PLGA and PBAE, several additional polymers have been explored in combination with PEG-lipid for pulmonary mRNA delivery. A modular lipid–polymer hybrid nanoparticle system was developed by incorporating medium-chain-length polyhydroxyalkanoates (mcl-PHAs) as an alternative to PLGA in combination with either DMG-PEG or poly(2-ethyl-2-oxazoline)-myristic acid (PEtOx-MA). Importantly, the biophysical properties and transfection efficacy of these nanoparticles were preserved following lyophilization and storage across a range of temperatures for up to two months. Reporter mRNA expression was detected across multiple organs following intravenous injection in mice [55]. Ionizable dendrimer-based nanomaterial incorporating lipid-anchored PEG have also been formulated with self-replicating RNA for in vivo antigenic RNA delivery. These dendrimer formulations elicited robust antibody responses, antigen-specific CD8^+^ T-cell responses, and protective immunity against lethal challenges with Ebola, influenza, and Zika viruses [56]. An optimized one-component ionizable amphiphilic Janus dendrimers (IAJD) was further employed as a delivery vehicle for anti-inflammatory TGF-β mRNA to the lungs, demonstrating efficacy in attenuating pulmonary inflammation [57]. Additional polymeric strategies include mRNA-loaded nanoparticles based on diethylenetriamine-substituted poly(aspartic acid) (P[Asp(DET)]) which support pulmonary protein expression with tissue targeting modulated by optimized surface PEG density [58].

### 4.3. Lipid-Extracellular Vesicle (EV) Hybrid Nanoparticles

Extracellular vesicles (EVs) represent a compelling platform for mRNA mucosal delivery due to their intrinsic biological activity, low immunogenicity, and flexibility. Electroporation and lipid-mediated transfection are the most widely used methods for loading mRNA into EVs [59,60]. A microfluidic electroporation approach has been introduced to generate IFN-γ mRNA-loaded, CD64-overexpressing EVs [61]. An acoustic shock wave-based method was employed to load either protein or mRNA antigens into EVs derived from LPS-activated THP-1 monocytes, achieving approximately 75% encapsulation efficiency for SARS-CoV-2 receptor-binding domain (RBD) mRNA. Immunization with these mRNA-EV vaccines induced potent humoral and balanced Th1/Th2 cellular immune responses. Notably, lyophilized formulations retained immunogenicity for up to seven days at 4 °C, supporting cold-chain-independent distribution [62]. Despite these advances, efficient mRNA loading and functional intracellular delivery remain significant technical challenges to EV-based RNA platforms.

To overcome these limitations, a hybrid EV platform (HEVs) was developed by fusing EVs with LNPs in MES buffer at pH 5.5. This approach preserved classical EV characteristics while enhancing mRNA encapsulation efficiency and promoting endosomal escape, thereby enabling more effective intracellular delivery. HEVs demonstrated favorable tolerability and extrahepatic distribution, underscoring their potential as a versatile mRNA delivery platform [63]. In a parallel effort, EV-mimetic cell-derived nanovesicles (CNVs) were co-formulated with conventional LNP lipid components via microfluidic mixing. The resulting hybrid EV nanoparticles (CELLNPs) exhibited significantly higher cellular uptake relative to LNP controls, though endosomal escape efficiency and protein expression levels remained comparable. While CELLNPs outperformed EVs passively loaded with endogenous mRNA via LNP transfection, HEVs achieved the highest proportion of functionally active hybrids capable of delivering mRNA to recipient cells, surpassing CELLNPs in hybrid formation efficiency [64].

Beyond immune applications, the therapeutic scope of lipid–EV hybrids has been explored. Given the well-established regenerative properties, stem cell-derived EVs were selected for hybrid nanoparticle development. Specifically, mesenchymal stem cell-derived EVs (MSC-EVs), recognized for their pro-regenerative characteristics and utility as drug delivery vehicles, were incorporated into MSC-EV-derived hybrid lipid nanoparticles (MSC-Hyb NPs) using a microfluidic-sonication technique. This platform was engineered for the delivery of collagen type I (COL1A1) mRNA into pathological tendon stem/progenitor cells (TSPCs), demonstrating the therapeutic versatility of EV-LNP hybrids beyond vaccine-oriented applications [65]. In another study, milk-derived EV-LNP hybrids were explored for oral RNA delivery. Compared with conventional LNPs, these hybrids exhibited lower cytotoxicity, altered epithelial uptake pathways, and markedly improved intestinal epithelial transport, broadening the applicability of EV-LNP systems to mucosal routes beyond the respiratory tract [66].

Alongside hybrid approaches, native EVs have also demonstrated considerable promise as both delivery vehicles and adjuvants for intranasal vaccination. Intranasal administration of influenza HA-conjugated EVs elicited robust HA stalk- and virus-specific antibody responses accompanied by enhanced mucosal immunity, conferring complete protection against heterologous influenza infection in mice [67]. Mosaic HA-EV vaccines co-displaying HAs from two distinct influenza groups induced broad cross-reactive antibody responses and cellular immunity, conferring protection against heterosubtypic reassortant H7N9 and H5N1 infections [68]. EVs derived from diverse cell sources, including 293T, MDCK, A549, and mature bone marrow-derived dendritic cells (mDC-EVs), have all demonstrated potent mucosal adjuvanticity [67,69].

Taken together, EV-based RNA platforms represent a highly promising and versatile direction for respiratory mucosal mRNA delivery, encompassing both vaccine development and pulmonary disease therapeutics. The inherent tunability of both EV and LNP engineering components enables the rational design of optimized formulations with reduced toxicity, enhanced cargo expression, and improved tissue targeting. As the field advances, hybrid EV-LNP platforms hold considerable potential to confer durable protective mucosal immunity against respiratory viral infections and to offer effective therapeutic strategies for pulmonary diseases.

**Figure 1:**
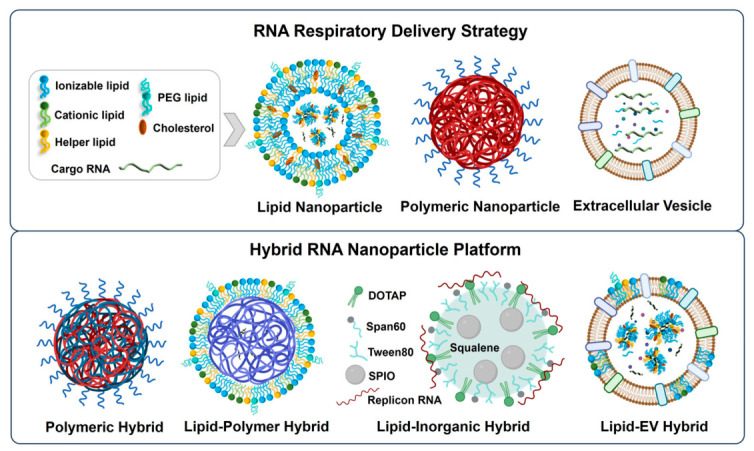
Schematic illustration of RNA delivery strategies. Lipid nanoparticles (LNPs), polymer-based nanoparticles, and extracellular vesicles (EVs) represent promising platforms for respiratory RNA delivery (upper panel). To further enhance delivery efficiency and immunogenicity, various hybrid RNA nanoplatforms have been developed, including polymeric hybrid nanoparticles, lipid–polymer hybrid nanoparticles, lipid-inorganic hybrid nanoparticles, and lipid–EV hybrid nanoparticles (lower panel).

Overall, hybrid nanoparticle platforms exhibit distinct advantages depending on their composition, offering a promising strategy to integrate complementary features of lipid, polymer, and biological delivery systems for mRNA vaccination. LIONs offer high structural stability, scalable manufacturing, and strong immunogenicity, making them attractive for clinical translation, although their cationic components may induce inflammatory responses. In contrast, lipid-polymer hybrids provide greater flexibility in tuning release kinetics, mucus penetration, and tissue targeting, but require more complex formulation optimization and may suffer from batch-to-batch variability. EV-based hybrid systems uniquely benefit from intrinsic biocompatibility and adjuvanticity, yet remain limited by challenges in scalable manufacturing and reproducible mRNA loading efficiency. Collectively, these platforms demonstrate complementary strengths while sharing unresolved challenges in balancing delivery efficiency, safety, and manufacturing feasibility, underscoring the need for unified evaluation criteria to enable meaningful comparison across platforms. Key features of the mRNA nanoparticle systems discussed in this review are summarized in Table 1 and Table 2, and representative delivery platforms are illustrated in Figure 1.

## 5. Optimization of RNA Nanoparticle Platform

### 5.1. Adjuvants

Although mRNA-LNP platforms possess intrinsic self-adjuvanticity, incorporation of exogenous adjuvants offers a promising approach to overcome immune tolerance in mucosal tissues [70,71]. TLR agonists such as CpG and Poly I:C share physicochemical properties with RNA that facilitate their seamless integration into mRNA nanoparticle formulations [21]. Similarly, the STING agonist cGAMP has been widely investigated as a mucosal adjuvant in combination with lipids and polymeric nanoparticles. cGAMP encapsulated within pulmonary surfactant mimetic liposomes (PS-GAMP) was employed as an adjuvant in an intranasal influenza vaccine, inducing lung-resident memory CD8^+^ T cells and conferring cross-protection against different influenza strains [72]. An RBD nanoparticle vaccine assembled from mannan, polyarginine, and cGAMP elicited strong mucosal and systemic immunity following intranasal administration, conferring protection against SARS-CoV-2 challenge [73]. Interestingly, a nanoparticle STING agonist liposome (NanoSTING) composed of DPPC, DPPG, cholesterol, DPPE-PEG2000, and cGAMP functioned as a potent immune activator in mice, hamsters and nonhuman primates, providing protection against SARS-CoV-2 in multiple animal models [74].

Beyond small-molecule adjuvants, co-encapsulation of immunostimulatory mRNA constructs within LNPs represents a particularly flexible and translationally attractive strategy. Cytokine-encoding mRNAs, including GIFT4 and CCL27, have been co-formulated with antigen-encoding mRNA in LNPs to enhance humoral and systemic T-cell responses, promote early germinal center reactions, and confer cross-protection against heterologous influenza strains [75]. Likewise, co-delivery of IL-12 mRNA-LNPs augmented CD8^+^ T-cell expansion, effector function, and tissue-resident memory formation, improving protection in both infectious disease and tumor model [76]. In a recent study, an mRNA-encoded antigen was fused to a C3 complement-derived adjuvant and co-formulated with an optimized biodegradable ionizable lipid bearing cyclic amine headgroups. This multi-adjuvanted strategy elevated anti-SARS-CoV-2 antibody titers by tenfold compared to unadjuvanted formulations following both intramuscular and intranasal administration [77]. Together, these approaches highlight the versatility of integrating adjuvants into mRNA nanoparticle platforms to fine-tune and amplify protective immune responses.

### 5.2. PEG Alternatives

**Figure 2:**
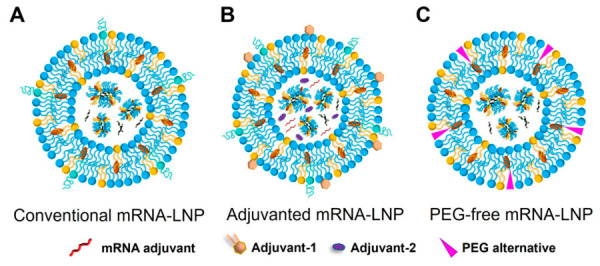
Optimized mRNA-LNP platforms. (**A**) Conventional mRNA-LNP. (**B**) Schematic of mRNA-LNPs containing adjuvants encapsulated within the nanoparticle core or displayed on the lipid membrane surface. (**C**) mRNA-LNPs formulated with PEG alternatives.

PEG-lipids play a crucial role in nanoparticle formation, mucus-penetration and modulation of immune response. However, PEG has been associated with immunogenic reactions, including the production of anti-PEG antibodies, that may cause adverse effects and prevent repeat dosing, therefore PEG alternatives are being developed for RNA therapeutics [78,79]. LNPs containing brush-shaped poly (ethylene glycol) methyl ether methacrylate (PEGMA) lipids (BPLs) exhibited higher transfection efficiency than conventional DMG-PEG2000-containing LNPs and effectively blocked anti-PEG antibody binding [80]. Poly(carboxybetaine) (PCB) lipid-containing LNPs have demonstrated higher mRNA transfection efficiency than PEG-containing LNPs across different formulations, with enhanced endosomal escape. These formulations also exhibited a favorable immunotoxicity profile and effectively mitigated the accelerated blood clearance observed for PEG-containing LNPs, enabling repeated administration without efficacy loss [81]. Additional PEG alternatives currently under investigation include poly(N-methyl-N-vinylacetamide) (PNMVA), polyoligo(ethylene glycol) methyl ether methacrylate (POEGMA), poly(*N*-(2-hydroxypropyl) methacrylamide) (PHPMA), and poly(N,N-dimethylacrylamide) (PDMA) [82], as well as their derivatives-DSPE-PNMVA, POEGMA-DSG, PHPMA-DSG, and PDMA-DSG [83,84]. The application of PEG alternatives within RNA nanoparticle formulations specifically designed for respiratory delivery remains relatively underexplored. Further investigation in this area is expected to yield valuable insights into the rational optimization of RNA nanoparticles for lung delivery and the induction of protective immune response against respiratory viral infections. The optimized mRNA-LNPs incorporating adjuvants or PEG alternatives were shown in Figure 2.

### 5.3. Delivery Strategies

Heterologous vaccination approaches have emerged as a promising strategy to overcome the limitations of conventional homologous regimens and to augment both systemic and mucosal immune responses. Numerous studies have demonstrated that heterologous prime-boost strategies employing parenteral priming followed by intranasal boosting enhance protection against respiratory viral infections across distinct vaccine platforms and antigen formulations [85,86,87,88]. Integration of advanced mRNA nanoparticle systems into heterologous mucosal vaccination strategies may further promote the establishment of long-term lung-resident cellular immunity.

Inhalation-based delivery of mRNA formulation has emerged as a promising modality for mucosal vaccination, enabling direct deposition of mRNA nanoparticles throughout the respiratory tract and facilitating local antigen expression within the lungs [15,16]. Compared with invasive intratracheal and intranasal administration, nebulized aerosols may achieve deeper pulmonary deposition, broader airway distribution, and prolonged interaction with mucosal immune cells. Nevertheless, nebulized mRNA formulations face challenges related to stability during aerosolization and penetration across respiratory mucosal barriers. Optimized mRNA systems—engineered through rational selection of ionizable lipids, refined lipid composition and PEG molar ratios, and incorporation of polymeric excipients—have demonstrated improved stability during aerosolization with enhanced RNA delivery to the lung [15,16,18,19]. These advanced mRNA formulations hold promise for integration into heterologous vaccination strategies to induce robust and durable mucosal immune responses.

## 6. Conclusions

In conclusion, substantial advances in RNA nanoparticle engineering have significantly expanded the potential of respiratory mucosal delivery for mRNA vaccines and therapeutics. Rational design of lipid nanoparticles, polymer-based carriers, and hybrid systems can improve mRNA stability, mucus penetration, pulmonary targeting, and antigen delivery efficiency in the complex respiratory environment. In addition, complementary strategies such as adjuvant incorporation, PEG alternatives, and heterologous immunizations may further strengthen both mucosal and systemic immune responses, enabling more effective immunological activation at the primary site of pathogen invasion. Together, these developments highlight the versatility of RNA nanoplatform engineering in controlled delivery and shaping functional immune responses in respiratory tissues. The growing understanding and investigation of RNA nanotechnologies and their associated immune responses will establish powerful and rapid advancing platforms for respiratory RNA delivery, supporting the translational applications in vaccination and pulmonary therapeutics.

## 7. Challenges and Future Perspectives

Looking ahead, several key design principles are expected to advance mRNA nanoparticle platforms for respiratory mucosal vaccination. One objective is the development of rational targeting strategies that direct nanoparticles toward immunologically relevant cell populations within mucosal tissues, including nasal epithelial cells, dendritic cells (DCs), alveolar macrophages and M cells. Surface engineering of LNPs or EVs with cell-specific ligands, antibodies, or targeting peptides may further enhance selective cellular uptake. Concurrently, the development of mucus-penetrating nanoparticles remains a critical challenge for effective mucosal delivery, as the mucus barrier substantially restricts nanoparticle diffusion and access to target cells. Potential strategies include the incorporation of muco-inert surface coatings, PEG alternatives, or charge-shielding designs that facilitate nanoparticle diffusion through mucus while maintaining cellular uptake. In addition, controlled release of mRNA cargo is increasingly recognized as an important factor for immune programming, as prolonged antigen expression has been shown to promote the induction and maintenance of tissue-resident memory T cells, a population essential for durable mucosal protection. Such controlled antigen expression may be achieved through optimized nanoparticle composition, sustained-release formulations, or self-amplifying RNA platforms. Beyond biological barriers, clinical translation of respiratory mRNA nanoparticle platforms will require addressing additional challenges, including formulation stability during storage and aerosolization, scalable and reproducible manufacturing, and regulatory requirements for quality control and safety evaluation. These considerations will be critical for advancing promising preclinical platforms toward clinical application.

In addition to these development-related challenges, safety considerations specific to respiratory delivery routes represent a critically translational barrier. Although significant advances in respiratory mRNA nanoparticle delivery, safety considerations specific to intranasal and pulmonary administration remain incompletely standardized. In addition to inflammation and tolerability concerns, route-specific risks such as potential nose-to-brain transport via the olfactory region warrant careful evaluation, particularly in the context of repeat or long-term intranasal dosing. Repeated aerosol or intranasal exposure may also induce localized epithelial irritation and cumulative inflammatory responses, which are not consistently assessed across preclinical studies. A further limitation is the lack of standardized safety and tolerability endpoints, such as cytokine profiling, bronchoalveolar lavage fluid analysis, and histopathological scoring, limiting direct cross-study comparison. Collectively, these factors highlight the need for standardized, route-specific safety evaluation frameworks to support the clinical translation of respiratory mRNA nanoparticle platforms.

Across the respiratory mRNA nanoparticle platforms, another challenge is balancing delivery efficiency with pulmonary tolerability. While moderate increases in cationic charge can enhance mRNA encapsulation, mucus penetration, cellular uptake, and endosomal escape, thereby improving transfection efficiency. However, these same physicochemical properties may also increase cytotoxicity, induce local inflammatory responses, and reduce respiratory tract tolerability, particularly in the context of mucosal administration. Consequently, optimizing the balance between potency and safety remains a fundamental design principle in the field. Emerging strategies, including biodegradable ionizable materials, charge-shielding approaches, biomimetic surface modifications, and hybrid nanoparticle systems, are being actively explored to preserve delivery efficacy while minimizing adverse pulmonary effects.

From a translational perspective, LNP-based systems remain the most clinically advanced platform, whereas EV-based and hybrid systems are still in early translational stages due to manufacturing and mechanistic limitations. Emerging evidence indicates that EVs are potent natural immunomodulators capable of activating innate immunity, enhancing antigen uptake, and shaping mucosal immune response. Specific EV-associated proteins, lipids, and glycans function as endogenous pulmonary immunoregulators that promote protective innate activation while limiting excessive lung inflammation. Systemic investigation of these EV-associated immunomodulatory components may inform the identification and development of novel mucosal adjuvants for respiratory mRNA vaccines. In addition, polymer-based mRNA nanoparticle systems remain largely in the preclinical stage. Although they demonstrate promising immunogenicity and formulation flexibility, their clinical translation is currently limited by challenges in safety, in vivo stability, reproducibility, and scalable manufacturing. Importantly, clinical translation will require clearly defined success criteria, including robust mucosal and systemic immunity, durability of protection, safety in the respiratory tract, and potential reduction in viral transmission, together with scalable GMP manufacturing and regulatory feasibility.

Collectively, integrating precise cellular targeting, mucus-penetrating surface engineering, and controlled antigen release into next-generation hybrid mRNA nanoparticle platforms may provide a promising strategy for inducing broad, robust, and long-lasting protective mucosal immunity against respiratory viral infections.

## Figures and Tables

**Table 1:** mRNA nanoparticle platforms for respiratory mucosal immunity.

Modified Lipid Nanoparticles (LNPs)					
Ref.	Platform	Key Design	Cargo	Route/Application type	Features
[20]	Ionizable mRNA-LNP	Ionizable lipids (ALC-0315, SM-102, MC3) ± cationic lipid (DOTAP/DOTMA)	mRNA	IN/Vaccine	Lung epithelial and immune cell transfection; limited mucosal immunogenicity; recalls draining lymphoid tissue responses
[21]	Charged-helper lipid LNP	Standard helper lipids replaced with neutral, anionic, or cationic lipids	mRNA	IV	Non-hepatocellular delivery; redirected lung and spleen tropism
[22]	Lung-targeting LNP	Supplemental cationic lipid added to ionizable LNP formulation	mRNA	IV	Significantly increased lung-selective mRNA delivery
[23]	Ionizable lipid-like NP (iLLN)	Ionizable + cationic lipid hybrid; liquid-core; triolein + beta-sitosterol; PEGylated muco-inert surface	mRNA	IN/Vaccine	pKa matched to nasal pH; ~60-fold higher nasal gene expression; enhanced mucus penetration and tolerability
[29]	Inhalable PEGylated LNP	Combination of β-sitosterol and high PEG contents, aerosol-stable ionizable LNP	mRNA	Inhalation	Rapid mucus penetration; improved lung epithelium transfection; PEG >5% reduces transfection efficiency
[30]	Cationic cholesterol LNP	Cationic cholesterol + cationic helper lipids	mRNA	IV	mRNA delivery to lung epithelial, stem-like, and cardiac cells; distinct biodistribution vs. conventional LNPs
[31]	SARS-CoV-2 mRNA-LNP	Spike mRNA in ionizable LNP	mRNA	IN/Vaccine	Successful immunogenicity and protective efficacy in hamster challenge model
Polymer-Based mRNA Nanoparticles					
Ref.	Platform	Key Design	Cargo	Route/Application type	Features
[32]	PEI/saRNA polyplex	Large excess of the cationic PEI and single saRNA molecules in solution	saRNA	IM	Small spherical NP with high packing density and low polymer mass fraction; PEI/HA-saRNA induced immune responses in mice
[33]	PBAE-PEG-lipid NP	Degradable PBAE terpolymers + PEG-lipid conjugate	mRNA	IV	Increased serum stability and in vitro potency; functional delivery of mRNA to lung
[34]	Hyperbranched PBAE (hPBAE) polyplex	hDD90-118 and hC32-118 hPBAE mRNA polyplex	mRNA	Inhalation	Consistent protein expression in lung epithelium with minimal toxicity
[35]	hPBAE/Cas13a mRNA NP	hDD90-118 co-delivering Cas13a mRNA + guide RNA; nebulized	mRNA	Inhalation/Therapeutic	Reduces SARS-CoV-2 replication in mice and hamsters via inhaled CRISPR-Cas13a
[36]	Optimized Polymer–lipid NP	PBAE + PEG-lipid hybrid; tailored polymer synthesis	mRNA	IV	Targeted delivery to lung endothelium and pulmonary immune cells
[38]	PBATE P76 polymer NP	Poly(β-amino-thio-ester) (PBATE) P76; nebulized; species-agnostic	mRNA	Nebulization/Therapeutic	Active in mice, hamsters, ferrets, cows, and NHPs; Fourfold dose sparing vs. prior PBAEs; favorable safety profile
[39]	PBAE NP library screen	PBAE NPs with varying hydrophobic monomer content; high-throughput image-based screen	mRNA	IV	High lung expression across most formulations; safe and efficacious mRNA expression in multiple tissues
[40]	PBAE mRNA NP	Bioreducible PBAE co-delivering CpG ODN or Poly (I:C) + antigen mRNA	mRNA + adjuvant	IV/Vaccine	Robust antigen-specific CD8^+^ T-cell responses; Splenic DC targeting
[41]	PACE mRNA NP	Optimized PACE with amine end-groups and PEG content	mRNA	IN/Intratracheal instillation/Vaccine	Protective mucosal immunity in rodents; safe profile; internalized by lung epithelial cells and APCs
[42]	PACE NP + RBC hitchhiking	PACE60 NPs adsorbed onto RBCs prior to administration	mRNA	IV	Enhanced lung delivery via RBC hitchhiking strategy
[43]	Hyperbranched PACE NP	Hyperbranched PACE (HBPA-E)	mRNA	Pulmonary/intranasal	Higher pulmonary and intranasal delivery efficiency than linear PACE; lyophilizable
[49]	PLGA/PBAE hybrid NP	FDA-approved PLGA + PBAE hybrid; vibrating-mesh nebulization compatible	mRNA	Nebulization/Vaccine	Mucus penetration; ex vivo human lung transfection; superior post-nebulization activity vs. SM-102 LNP
[51]	PLGA/PEI NP	PLGA core + PEI/PLL cationic coating	mRNA	In vitro	Efficient dendritic cell transfection
[58]	P(Asp(DET)) NP	Diethylenetriamine-substituted poly(aspartic acid); PEG-tuned surface	mRNA	IM	Pulmonary protein expression; tissue targeting modulated by PEG surface coverage
Lipid–Polymer Hybrid Nanoparticles					
Ref.	Platform	Key Design	Cargo	Route/Application type	Features
[53]	PLGA-TT3 lipid–polymer NP	PLGA core + TT3 ionizable lipid + DOPE + Cholesterol + DMG-PEG2000	mRNA	In vitro	Enhanced mRNA delivery vs. Intravenous TT3-only LNP
[54]	PLGA-C12-200 lipid–polymer NP	PLGA core + C12-200 ionizable lipid + DOPE + DMPE-PEG2000	mRNA	IM/Vaccine	Spike-specific antibody and CD8^+^ T-cell responses; inhibited SARS-CoV-2 in hamsters
[55]	mcl-PHA lipid–polymer NP	Medium-chain-length PHA (mcl-PHA) + DMG-PEG or PEtOx-MA	mRNA	IV	Preserved efficacy after lyophilization; multi-organ expression after IV injection; lyophilizable
[56]	Dendrimer-RNA NP	Ionizable dendrimer + lipid-anchored PEG formulated with repRNA	repRNA	IM/Vaccine	Elicit both CD8^+^ T-cell and antibody responses; protective immunity against lethal Ebola, influenza, and Zika challenges
[57]	Dendrimer-TGF-β mRNA	Lung-specific IAJD34; TGF-β delivery	mRNA (TGF-β)	IV/ Therapeutic	Protein expression in the lower regions of the lung; reduce inflammation in ITB model
Replicon RNA/Lipid-Inorganic Hybrid Nanoparticles					
Ref.	Platform	Key Design	Cargo	Route/Application type	Features
[44]	LION/repRNA-SARS-CoV-2	Cationic squalene emulsion (Span60/DOTAP/Tween80) embedding SPIO; repRNA assembled on surface via electrostatic interaction	repRNA (spike S)	IM/Vaccine	Robust antibody and T-cell responses in mice and NHPs
[45]	LION/repRNA-multi-antigen	LION with repRNA encoding multi-component antigens	repRNA (multi-antigen)	IM/Vaccine	Robust antibody responses with reduced systemic inflammation vs. LNPs in pigtail macaques
[46]	Lyophilized LION/repRNA	LION/repRNA lyophilized with 10% sucrose cytoprotectant	repRNA	IM/Vaccine	Stable at 2–8 degrees; preserved antigen expression and antibody responses vs. liquid formulation
Lipid-Extracellular Vesicle (EV) Hybrid Nanoparticles					
Ref.	Platform	Key Design	Cargo	Route/Application type	Features
[61]	mRNA-loaded EV	Microfluidic electroporation loading IFN-γ mRNA into CD64-overexpressing EVs	mRNA (IFN-γ)	IV/ Therapeutic	Targeted mRNA delivery for cancer immunotherapy
[62]	Monocyte-EV mRNA vaccine	Acoustic shock wave loading of RBD mRNA into LPS-activated THP-1 EVs	mRNA	IM/Vaccine	~75% encapsulation; lyophilizable; potent humoral and balanced Th1/Th2 responses; stable lyophilized form
[63]	Hybrid EV (HEV)	EV-LNP fusion in MES buffer (pH 5.5), PH controlled	mRNA	IV	Enhanced mRNA encapsulation; endosomal escape; extrahepatic delivery; predominant functional distribution in spleen
[64]	CELLNPs	Microfluidic mixing of hiPSC-EVs with lipid mix	mRNA	In vitro	Low hybridization efficiency; increased uptake; comparable endosomal escape and protein expression compared to LNP
[65]	MSC-EV-LNP hybrid	MSC-derived EVs integrated into hybrid LNPs via microfluidic sonication	mRNA	In vitro	COL1A1 mRNA delivery to tendon stem/progenitor cells; therapeutic EV-LNP
[66]	Milk-EV-LNP hybrid	Milk-derived EVs fused with siRNA loaded LNP by microfluidic micromixer	siRNA	Oral gavage	Lower cytotoxicity; improved intestinal epithelial transport vs. LNPs; preferential colonic accumulation

Abbreviations: LNP, lipid nanoparticle; NP, nanoparticle; PBAE, poly(β-amino ester); hPBAE, hyperbranched PBAE; PBATE, poly(β-amino-thio-ester); PACE, poly(amine-co-ester); PLGA, poly(lactic-co-glycolic acid); PEI, polyethyleneimine; PLL, poly-L-lysine; EV, extracellular vesicle; LION, lipid-inorganic nanoparticle; SPIO, superparamagnetic iron oxide; sa/repRNA, self-amplifying/replicon RNA; APC, antigen-presenting cell; DC, dendritic cell; NHP, nonhuman primate; RBC, red blood cell; MSC, mesenchymal stem cell; HA, hemagglutinin; CELLNPs, cell-based lipid nanoparticles; hiPSC-EVs, human-induced pluripotent stem cell EVs; IV, intravenous; IM, intramuscular; IN, intranasal; ionizable amphiphilic Janus dendrimer (IAJD); intratracheal bleomycin (ITB) model.

**Table 2:** Comparative preparation methods, physicochemical properties, and biocompatibility of mRNA nanoparticle platforms for respiratory mucosal delivery.

Platform	Preparation Method (General)	Size *	Key Physicochemical/Functional Properties	Biocompatibility & Translational Status
Lipid Nanoparticles	Microfluidic mixing of ionizable lipids, helper lipids, cholesterol, and PEG-lipids; tunable lipid composition	~60–150 nm	High mRNA encapsulation; tunable ionization; mucus-penetrating or lung-targeting via lipid modification [20,21,22,23,24,25,26,27,28,29,30,31]	Clinically validated systemic safety; respiratory delivery still limited; limited mucosal immunogenicity
Polymer-Based Nanoparticles	Self-assembly or nanoprecipitation of cationic/biodegradable polymers (PBAE, PEI, PACE, PLGA); variable synthesis	~50–200 nm (highly variable)	High structural tunability; strong nucleic acid binding; adjustable degradation kinetics and surface charge [32,33,34,35,36,37,38,39,40,41,42,43]	Generally favorable preclinical safety (variable by polymer); cytotoxicity concerns for polycationic polymers; limited clinical translation
Lipid–Polymer Hybrid Nanoparticles	Hybrid assembly combining polymer cores (PLGA/PBAE/PHA) with lipid/PEG surface components	~80–200 nm	Combined stability and transfection efficiency; improved mucus penetration; aerosol-stable formulations [49,50,51,52,53,54,55,56,57,58]	Improved biocompatibility vs. polymers alone; still preclinical for mucosal delivery applications
Lipid-Inorganic Nanoparticles (LION)	Self-assembly of lipid emulsions (squalene, Span60, DOTAP, Tween80) with inorganic cores (e.g., SPIO)	~70–200 nm	Enhanced stability; strong immunogenicity; lyophilizable formulations; preserved efficacy after storage [44,45,46]	Promising vaccine platforms with favorable immune activation; limited mucosal clinical data; thermostable formulations
Lipid–EV Hybrid Nanoparticles	Isolation from cells (ultracentrifugation, microfluidics) or fusion with LNPs via microfluidic mixing/electroporation/sonication	~50–150 nm	Natural membrane composition; microfluidic loading/fusion capability; high cellular uptake rates [61,62,63,64,65]	Improved biocompatibility; low immunogenicity; major limitations in manufacturing scalability and batch standardization

* Size ranges represent typical literature values for these platform types based on standard nanoparticle characterization.

## Data Availability

No new data were created or analyzed in this study. Data sharing is not applicable to this article.
